# Mid-Infrared Electro-Optical Modulation Using Monolithically Integrated Titanium Dioxide on Lithium Niobate Optical Waveguides

**DOI:** 10.1038/s41598-019-51563-5

**Published:** 2019-10-22

**Authors:** Tiening Jin, Junchao Zhou, Pao Tai Lin

**Affiliations:** 1 Department of Electrical and Computer Engineering, Texas A&M University, College Station, Texas, 77843 United States; 2 Department of Materials Science and Engineering, Texas A&M University, College Station, Texas, 77843 United States

**Keywords:** Mid-infrared photonics, Integrated optics

## Abstract

Tunable photonic circuits were demonstrated in the mid-Infrared (mid-IR) regime using integrated TiO_2_-on-LiNbO_3_ (ToL) waveguides. The upper waveguide ridge was made by a sputtered TiO_2_ thin film with broad transparency at λ = 0.4–8 µm and an optimized refractive index **n** = 2.39. The waveguide substrate is a z-cut single crystalline LiNbO_3_ (LN) wafer that has strong Pockels effect, thus enabling the tunability of the device through electro-optical (E-O) modulation. A sharp waveguide mode was obtained at λ = 2.5 µm without scattering or mode distortion found. The measured E-O coefficient **γ**_**eff**_ was 5.9 pm/V approaching **γ**_**31**_ of 8.6 pm/V of LN. The ToL waveguide showed a hybrid mode profile where its optical field can be modified by adjusting the TiO_2_ ridge height. Our monolithically integrated ToL modulator is an efficient and small footprint optical switch critical for the development of reconfigurable photonic chips.

## Introduction

Mid-IR photonic circuits have attracted a lot of attention because of their application in broadband optical communication and label-free biochemical sensing^1–3^. For the optical network, extending the present operational spectrum from the near-IR into the mid-IR region provides additional optical channels thus improving the data transmission rates^4^. Specifically, E-O devices capable of fast intensity modulation are essential components for high bit rate optical communication and information processing^5^. Previous works in mid-IR photonic circuits have been demonstrated in various silicon-based platforms, such as Si-on-insulator, Si-on-sapphire, Si-on-AlN, and pedestal Si^6,7^. However, silicon has low optical nonlinearity (χ^3^) and absence of E-O tunability from Pockets and Kerr effects that limit its application for nonlinear frequency conversion and high-speed optical signal modulation^8^. In addition, the present Si photonics platform utilizes SiO_2_ as the optical waveguide undercladding, while SiO_2_ becomes opaque at λ > 4.0 µm^9^. Thus, Si-on-insulator was unsuitable for broadband mid-IR applications.

Another material option to achieve reconfigurable mid-IR photonics is lithium niobate, a ferroelectric and high nonlinear optical crystal^10,11^. First, LN is transparent up to λ > 5 μm with a moderate refractive index, **n** = 2.18^12^, which grants it versatile roles either as a waveguide cladding or a waveguide core in the mid-IR regime^13,14^. Second, LN has a high E-O coefficient and a large second-order optical nonlinearity (χ^2^) so it enables efficient nonlinear light generation and E-O light modulation^15,16^. Previous studies have shown a LN switch in Near-Infrared (NIR) with a modulation speed exceeding 100 GHz^17^ and a low **V**_**π**_∙**L** of ∼10 Vcm^18^, where **V**_**π**_∙**L** is the product of the voltage and the device length to create a π phase difference. In addition, both phase and intensity LN modulators can achieve a high extinction ratio >15 dB, which is significantly better than present Si based photonic devices^19^. Therefore, LN is an ideal platform for tunable mid-IR photonic circuits.

In this work, we created reconfigurable waveguides that monolithically integrated ferroelectric LN and mid-IR transparent TiO_2_. From finite difference method (FDM) modeling, the ToL photonic circuits had a hybrid waveguide mode, where its optical field confined in the LN and the TiO_2_ layers can be optimized by the TiO_2_ thickness. Unlike the Si-on-LN waveguide that had its optical field dominantly confined in the high refractive index Si, our ToL device was able to confine majority of the lightwave in the ferroelectric LN layer, which is critical to achieve efficient E-O modulation. This is because TiO_2_ and LN have a lower refractive index contrast, **∆n** = 0.21, compared to a **∆n** = 1.3 between Si and LN. The ToL waveguide structures were characterized by a scanning electron microscope (SEM) equipped with energy-dispersive X-ray spectroscopy (EDS). Meanwhile, the effective E-O coefficient in mid-IR regime was obtained by measuring the mode intensity variation when an external electrical field **E** was applied across the waveguide. Hence, the developed ToL platform enables reconfigurable mid-IR photonic circuits desired for broadband optical communication.

## Experimental Methods

**Figure 1:**
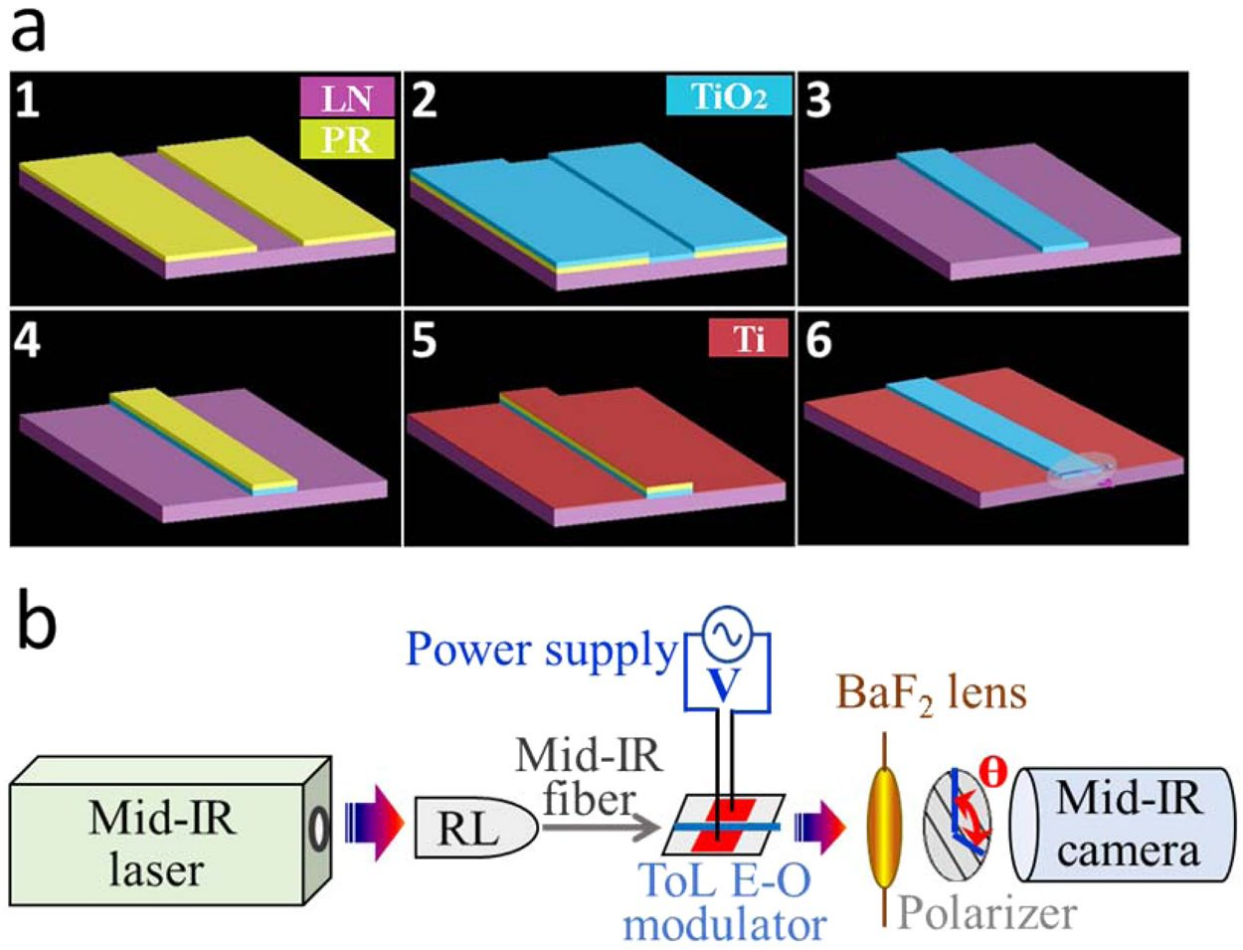
(**a**) Schematic of the ToL waveguide fabrication process. The waveguide structure was defined by photoresist (PR) on the LN substrate through photolithography, following TiO_2_ thin film deposition by reactive RF sputtering. The TiO_2_ ridge waveguide structure was developed on the LN substrate after lift-off. Ti electrodes were loaded on both sides of the waveguide through the same fabrication process. (**b**) The experimental set-up to test the e-o modulation of the ToL waveguide. Light from mid-IR laser was coupled into a fiber through the reflective lens (RL) and then into the ToL waveguide through butt-coupling technique. The electrodes were connected to a DC power supply and created the electric field E across the waveguide. The waveguide mode and intensity were recorded by a mid-IR camera. A polarizer with polarization angle ϴ was placed between the BaF_2_ lens and the camera.

The detailed device fabrication process is shown in Fig. 1a. First, a negative tone photoresist was patterned on a LN wafer, which defined the waveguide structure. After development, the photoresist had undercut sidewall that will facilitate the lift-off process. Next, a 0.8 µm thick TiO_2_ film was deposited on the patterned substrate by room temperature RF sputtering. The target was Ti (99.999%) and the gases introduced into the deposition chamber were 36 sccm argon and 4 sccm oxygen. After TiO_2_ deposition, the photoresist and the TiO_2_ above were removed by acetone and only the TiO_2_ ridge waveguide was left on the LN substrate. Applying the same lithography and lift-off steps, 100 nm thick Ti electrodes were deposited on both sides of the TiO_2_ waveguide. The ToL fabrication avoided delicate and costly processes including ion slicing and wafer bonding utilized in prior LN thin film devices^20,21^.

The waveguide optical property and the E-O reconfigurability of the ToL were characterized by a testing station shown in Fig. 1b. The light source was a pulsed laser with a wavelength tunable from λ = 2.4 to 3.8 μm and a linewidth of 3 cm^−1^. It had a 150 kHz pulse repetition rate, 10 nano seconds pulse duration, and 150 mW average power. The probe laser light was first focused into a 9 μm core fluoride fiber using a reflective lens, and then butt-coupled into the waveguide. The fine alignment between the optical fiber and the ToL waveguide was monitored by an upper microscope equipped with a long working distance 10x objective lens. The light emitted from the waveguide back facet was focused by a CaF_2_ biconvex lens with 25 mm focal length and then captured by a liquid nitrogen cooled 640 × 512 pixel InSb camera. A polarizer was placed before the camera, selecting the polarization of the recorded light signal. Meanwhile, an electric field **E** was applied across the ToL waveguide using the pair of Ti electrodes that were loaded on the two sides of the TiO_2_ ridge. The electrodes were connected with a DC power supply through two micro-manipulators. **E** was determined by the applied voltage, **V**, of the power supply and the gap width, **g**, between the pair of electrodes. The waveguide mode intensity was then recorded when the **V** was swept between 0 and 100 V.

## Results and Discussion

**Figure 2:**
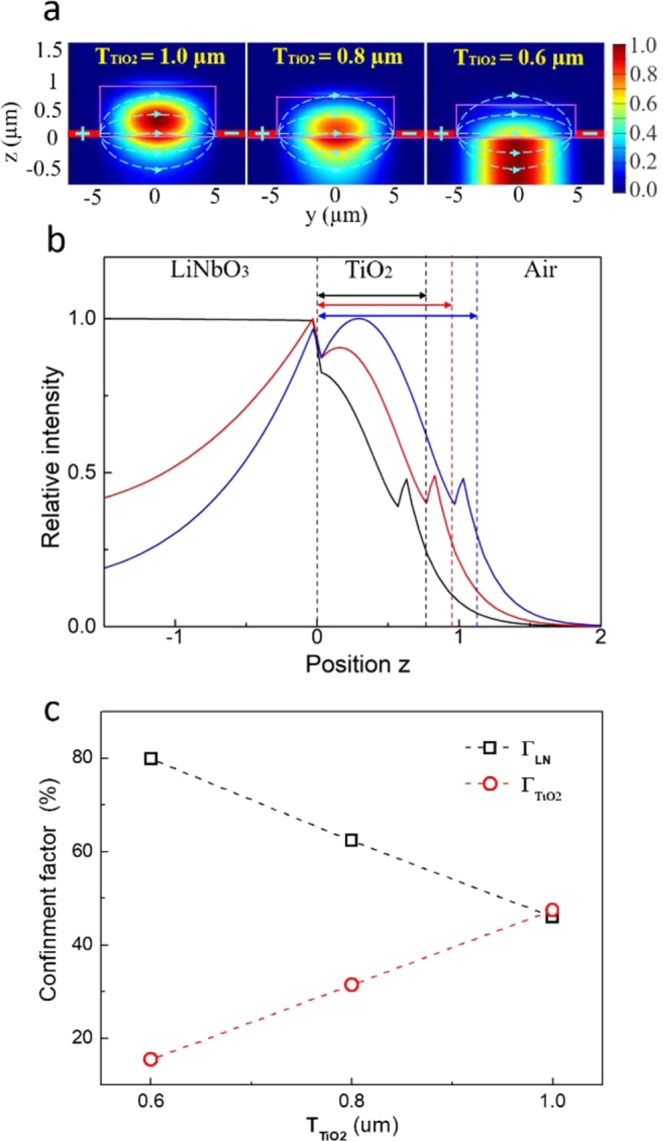
(**a**) The calculated optical field when the thickness of the TiO_2_ layer decreases from 1.0 µm to 0.6 µm. The center of the waveguide mode was shifted from the TiO_2_ layer to the LN layer. At **T**_**TiO2**_ = 0.8 µm, a hybrid waveguide mode was formed where the light was evenly confined in the TiO_2_ and the LN layers. (**b**) The calculated 1-D intensity profiles along the z direction at y = 0 µm, where the blue, red, and black curves indicate **T**_**TiO2**_ = 1.0, 0.8, and 0.6 µm, respectively. (**c**) The calculated optical field distribution factors, Γ_LN_ and Γ_TiO2_, at different T_TiO2_. Γ_LN_ increased and Γ_TiO2_ dropped as the TiO_2_ thickness increased.

The waveguide mode was numerically simulated by the two-dimensional FDM. In the modeling, a 12 μm × 6 μm TM light source was chosen to excite a mode at λ = 2.5 μm since its dimension is close to the 9 μm core mid-IR fiber used in the experiment. The refractive indexes, **n**, of TiO_2_ and LN are 2.39 and 2.18, respectively^22,23^. The mode profile and field intensity confined in the TiO_2_ and LN layers were modified by adjusting the TiO_2_ thickness, **T**_**TiO2**_. As shown in Fig. 2a at **T**_**TiO2**_ = 1 µm, a fundamental mode was clearly observed within the TiO_2_ ridge while a strong evanescent field appeared in the LN cladding layer, which was a characteristic profile belonging to the TM polarization mode^24^. As **T**_**TiO2**_ decreased to 0.8 µm, the mode center shifted toward the LN layer. A hybrid mode with a strong field existing in both LN and TiO_2_ layers was revealed. A ToL waveguide with this configuration is capable of efficient E-O modulation, since the optical field was confined in the ferroelectric LN layer and its overlapping with the electric field significantly increased. As **T**_**TiO2**_ decreased to 0.6 µm, no TM waveguide mode was found because the TiO_2_ layer was too thin to support a TM mode. To better visualize the variation of the optical field when **T**_**TiO2**_ changed, Fig. 2b shows the calculated 1-D intensity profiles parallel to the z direction at y = 0 µm. At **T**_**TiO2**_ = 1 µm, the intensity peak appeared in the center of the TiO_2_ layer. Meanwhile, the evanescent field in the lower LN cladding was much stronger than in upper air cladding since the LN has a higher refractive index than air. At **T**_**TiO2**_ = 0.80 µm, a hybrid mode was formed and the optical field in the ferroelectric LN layer became stronger, which was critical to achieve low **V**_**π**_∙**L** E-O modulation. On the other hand, at **T**_**TiO2**_ = 0.60 µm, there was no light confinement along the z direction. The TM waveguide mode disappeared since the TiO_2_ layer was too thin. The optical field distribution factors, **Γ**_**LN**_ and **Γ**_**TiO2**_, were defined as the percentages of optical fields found within the LN and the TiO_2_ layers, respectively. As shown in Table 1 and Fig. 2c, **Γ**_**LN**_ increased rapidly from 46.0% to 80.0% while **Γ**_**TiO2**_ decreased from 47.4% to 15.5%, as **T**_**TiO2**_ decreased from 1.0 to 0.60 µm. The optimized **T**_**TiO2**_ was 0.80 µm since it had a large **Γ**_**LN**_ and also a sufficient thickness to support the TM waveguide mode. Therefore, optimization of **T**_**TiO2**_ reshaped the intensity distribution of the waveguide mode, leading to a stronger optical field confined within the LN layer. Consequently, this enhanced the interaction between the mid-IR light wave and the electrical field that created high E-O reconfigurability and reduced optical propagation loss.

**Table 1:** The ToL waveguide’s optical field distribution factors, Γ_LN_, Γ_TiO2_, and Γ_Air_, at different thickness T_TiO2_. Γ_LN_ increased from 46.0 to 80.0 as T_TiO2_ decreased from 1.0 to 0.6 µm indicating an improvement of E-O modulation efficient.

T_TiO2_ (μm)	Γ_LN_ (%)	Γ_TiO2_ (%)	Γ_Air_ (%)
0.6	80.0	15.5	4.5
0.8	62.3	31.5	6.2
1.0	46.0	47.4	6.6

**Figure 3:**
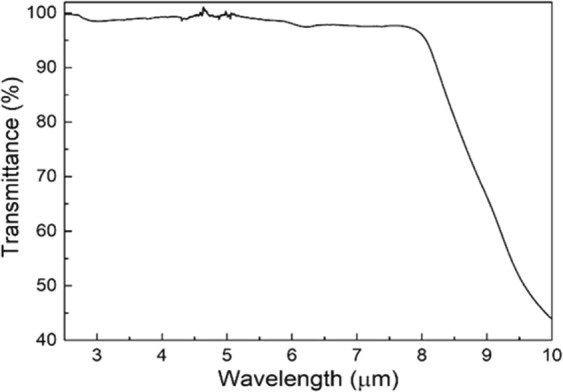
The transmission spectrum of deposited TiO_2_ from ATR-FTIR measurement, showing a broad transmittance up to λ = 8 µm.

The optical property of deposited TiO_2_ thin film was characterized by attenuated total reflection - Fourier transform infrared spectroscopy (ATR-FTIR) and the result is displayed in Fig. 3. High transmittance was found over a broad mid-IR spectrum between λ = 2.5 to 8.0 μm. The strong absorption after λ = 8.0 μm is due to the combinations of fundamental vibration modes existing at longer wavelengths, such as the Ti-O stretching and bending vibrations^25,26^. The results showed that the sputtered TiO_2_ thin film is a suitable material for mid-IR photonic circuits.

**Figure 4:**
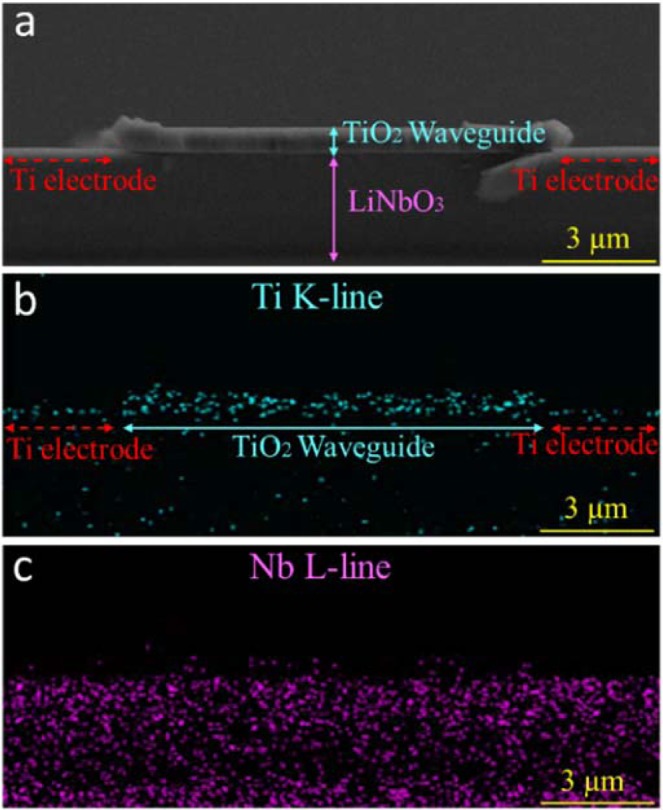
The cross-sectional view of the ToL waveguide from (**a**) SEM and EDX from (**b**) Ti K and (**c**) Nb L emission lines, indicating the TiO_2_ ridge, Ti electrodes, and the LN substrate, respectively. The TiO_2_ ridge is 0.8 µm thick and 10 µm wide. The waveguide surface and the interface between the TiO_2_ and LN was smooth. Ti electrodes were found next to the waveguide.

The structure of ToL device was inspected by a SEM equipped with EDS. Figure 4a shows the cross-sectional SEM image of a 10 μm wide and 0.8 μm height TiO_2_ ridge waveguide sitting on a LN substrate. Two Ti electrodes were loaded on each side of the waveguide. The device has a well-defined ridge structure without cracks or indents on the surface. The sharp waveguide edges reduced the waveguide propagation loss caused by light scattering. In addition, the interface between the TiO_2_ waveguide and LN layer was well-resolved. No depletion damage was found on the device surfaces or the interface because the waveguide was prepared by a lift-off process instead of an aggressive etching process. EDS with element mapping function was utilized to characterize the device layout and the material composition. As shown in Fig. 4b, the Ti profile identified the TiO_2_ ridge waveguide in the ToL center and two thin Ti electrodes loaded next to the waveguide. In Fig. 4c, the Nb distribution corresponded to the LN substrate that was underneath the TiO_2_ waveguide and Ti electrodes. The well-defined waveguide configuration and the high material uniformity avoided the optical loss caused by the variation of refractive indexes.

**Figure 5:**
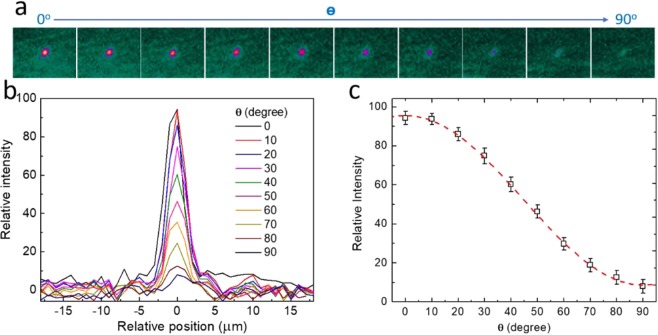
(**a**) The optical waveguide mode captured by an MIR camera when the polarizer rotated at different angle **ϴ**. The wavelength was set at λ = 2.5 µm. The mode decreased as **ϴ** changed from 0° to 90°. (**b**) The 1D intensity distribution of the waveguide mode recorded at various **ϴ**. A Gaussian mode profile was found. (**c**) The plot of the mode relative intensity vs the polarization angle **ϴ**. The red dash line is the fitting result using a cosine square function.

Figure 5a shows the optical waveguide mode at **T**_**TiO2**_ = 0.80 µm and its transmission at different polarizations. A sharp waveguide mode was clearly observed at λ = 2.5 µm when the polarization angle **ϴ** at 0° was parallel to the laser TM polarization. No scattering and distortion found in the captured images indicates that our waveguide has flat sidewalls and a smooth interface between the TiO_2_ and LN layer. As the **ϴ** rotated from 0° to 90°, the mode intensity dropped sharply indicating the ToL had a waveguide mode with TM polarization. The one-dimensional intensity distribution at different polarizations is drawn in Fig. 5b. The obtained Gaussian-like mode profile is similar to the hybrid mode resolved at Fig. 2b. Figure 5c shows the plot of mode intensity versus the **ϴ** and its fitting curve. The variation of intensity **I** with ϴ is consistent with the derived cosine squared function **I** (**ϴ**) = **I**_**0**_ cos^2^ **ϴ**, where **I**_**0**_ is the light intensity without a polarizer. At **ϴ** = 0°, a maximum transmission was obtained because the polarization of the waveguide mode was parallel to the axis of the polarizer. On the other hand, at **ϴ** = 90°, the transmission decreased to zero because the waveguide mode and the polarizer were cross polarized.

**Figure 6:**
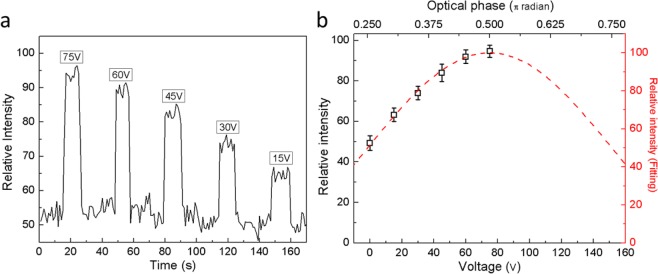
(**a**) The transient mode intensity when pulses of the electrical field were applied to the electrodes using a power supply at various voltage **V**. (**b**) The **I**-**V** plot extrapolated from transient response. The fitting result indicated by the red dash curve shows a cosine square function.

E-O analysis was performed by measuring the variation of the mode intensity when an external field was applied across the ToL waveguide. The polarizer was fixed at **ϴ** = 45° so the initial intensity was half of its maximum found at **ϴ** = 0°. The electric field **E** across the waveguide was adjusted between 0 to 7.5 V/µm by tuning the voltage **V** of the power supply. The transient mode intensity **I** was recorded and drawn in Fig. 6a. The intensity raised sharply whenever an electrical field was applied. Figure 6b displayed the **I**-**V** curve extrapolated from Fig. 5a and the fitting curve derived from the Pockels effect. The experimentally observed linear E-O response can be described by an equation as^27,28^

1 $$I(V)={I}_{0}[1-{\cos }^{2}({\phi }_{0}-\pi \frac{V}{2{V}_{\pi }})]$$

The parameter **V**_**π**_∙**L** = 50 V∙cm was found for the ToL waveguide. Through the equation^29,30^

2 $${V}_{\pi }=\frac{g\cdot \lambda }{2\cdot {\gamma }_{eff}\cdot {n}_{e}^{3}\cdot L\cdot {\Gamma }_{LN}}$$

the **γ**_**eff**_ was derived as 5.9 pm/V. The resolved **γ**_**eff**_ was slightly lower than the **γ**_**31**_ of 8.6 pm/V because the waveguide and the electrical field were not strictly parallel to the optical axis of the LN substrate. An even lower **V**_**π**_∙**L** of 7 V∙cm can be achieved if the electrode gap **g** is narrowed to 3 μm and the waveguide is aligned to the LN y-axis.

## Conclusion

Reconfigurable mid-IR photonic circuits were created using a monolithically integrated ToL platform. The device showed smooth waveguide sidewalls and a sharp TiO_2_ - LN interface because the waveguide was formed through the lift-off process instead of aggressive etching. After adjusting the thickness of the TiO_2_ layer, a hybrid waveguide mode with a large field confinement can be achieved at **T**_**TiO2**_ = 0.80 µm due to an optimized **∆n** of 0.21 between TiO_2_ and LN. The E-O tunability of the ToL waveguide was then realized by applying the Pockels effect from the LN substrate, where a **γ**_**eff**_ of 5.9 pm/V, close to **γ**_**31**_, was obtained at λ = 2.5 µm. The ToL waveguides provides efficient E-O modulation covering the mid-IR regime thus enabling broad spectrum optical communication.
